# The application of crowdsourcing approaches to cancer research: a systematic review

**DOI:** 10.1002/cam4.1165

**Published:** 2017-09-29

**Authors:** Young Ji Lee, Janet A. Arida, Heidi S. Donovan

**Affiliations:** ^1^ Department of Health and Community Systems School of Nursing University of Pittsburgh Pittsburgh Pennsylvania; ^2^ Department of Biomedical Informatics School of Medicine University of Pittsburgh Pittsburgh Pennsylvania; ^3^ Department of Obstetrics, Gynecology, and Reproductive Sciences School of Medicine University of Pittsburgh Pittsburgh Pennsylvania

**Keywords:** Cancer/neoplasm, citizen science, citizen scientists, crowdsourced, crowdsourcing, diffusion of innovation

## Abstract

Crowdsourcing is “the practice of obtaining participants, services, ideas, or content by soliciting contributions from a large group of people, especially via the Internet.” (Ranard et al. J. Gen. Intern. Med. 29:187, 2014) Although crowdsourcing has been adopted in healthcare research and its potential for analyzing large datasets and obtaining rapid feedback has recently been recognized, no systematic reviews of crowdsourcing in cancer research have been conducted. Therefore, we sought to identify applications of and explore potential uses for crowdsourcing in cancer research. We conducted a systematic review of articles published between January 2005 and June 2016 on crowdsourcing in cancer research, using PubMed, CINAHL, Scopus, PsychINFO, and Embase. Data from the 12 identified articles were summarized but not combined statistically. The studies addressed a range of cancers (e.g., breast, skin, gynecologic, colorectal, prostate). Eleven studies collected data on the Internet using web‐based platforms; one recruited participants in a shopping mall using paper‐and‐pen data collection. Four studies used Amazon Mechanical Turk for recruiting and/or data collection. Study objectives comprised categorizing biopsy images (*n* = 6), assessing cancer knowledge (*n* = 3), refining a decision support system (*n* = 1), standardizing survivorship care‐planning (*n* = 1), and designing a clinical trial (*n* = 1). Although one study demonstrated that “the wisdom of the crowd” (NCI Budget Fact Book, 2017) could not replace trained experts, five studies suggest that distributed human intelligence could approximate or support the work of trained experts. Despite limitations, crowdsourcing has the potential to improve the quality and speed of research while reducing costs. Longitudinal studies should confirm and refine these findings.

## Introduction

In the United States, the reach of cancer cannot be overstated: as of 1 January 2016, over 15.5 million Americans were living with a history of invasive cancer, and this number is projected to reach over 20 million by 2026 1. In response, both public and private institutions and organizations have devoted considerable financial resources to research that aims to understand the disease, develop treatment and interventions, and improve quality of life. For example, the 2015 fiscal year budget of the National Cancer Institute (NCI) was about $5 billion, an increase of $20 million over the previous fiscal year, and 42% of these funds were directed toward research grants 2. Nearly all clinical research involves recruiting an adequate number of participants to generate statistically meaningful findings; however, methodological challenges remain because recruitment is often a labor‐intensive and time‐consuming process 3.

As the number of Internet users continues to increase in the United States, with 84% of adults using the Internet in 2015 4, crowdsourcing has become a practical alternative to other, more traditional recruitment and/or outsourcing methods 5, 6, 7, 8. By definition, crowdsourcing is “the practice of obtaining participants, services, ideas, or content by soliciting contributions from a large group of people, especially via the Internet 9.” Initial applications of crowdsourcing in industry and commerce represented novel approaches to distributing burden; creative problem‐solving; idea‐generation and innovation; and knowledge sharing in areas as diverse as marketing, clothing design, astronomy, and journalism 10, 11. In recent years, these types of crowdsourcing methods increasingly have been adapted and applied to address a variety of problems in healthcare and healthcare research 12.

Healthcare research across the cancer continuum—from prevention to diagnosis to treatment, including the management of survivorship—could benefit from the application of crowdsourcing approaches to maximize efficiency while conserving resources. However, despite the growing use of these crowdsourcing approaches in healthcare research, the literature, to date, lacks any systematic reviews of the application of crowdsourcing approaches in cancer research. It is critical to comprehensively understand how crowdsourcing approaches can contribute to cancer research in order to fully benefit from these approaches in the future research. Therefore, our objective was to identify applications of crowdsourcing in cancer research and to explore the potential uses of this innovative strategy.

## Methods

A search of the PubMed, CINAHL, Scopus, PsychINFO, and Embase electronic databases was conducted in 2014 and repeated in July 2016 to locate studies of crowdsourcing applications in cancer research. Although PubMed, CINAHL, PsychINFO, and Embase index literature from the biomedical, behavioral, social science, nursing, and allied health fields, we also confirmed that Scopus retrieves studies from these disciplines as well as two major computer science databases, IEEE Explore and ACM Digital library. Including Scopus was therefore critical since crowdsourcing approaches have roots in the fields of computer and information science. The two search strings used for our search of the literature were the following: (1) *crowdsourced* OR *crowdsourcing* OR *citizen science* OR *citizen scientist* AND *cancer* OR *neoplasms*; (2) *crowdsourcing* OR *crowdsourced* OR *social networking* OR *diffusion of innovation* AND *cancer* OR *neoplasms*. Potentially relevant articles, based on their titles and abstracts, were independently evaluated by the first and second authors (YL and JA) to determine their eligibility for inclusion in the review.

Articles were included in our review if they met the following inclusion criteria: (1) peer‐reviewed studies (2) describing application(s) of crowdsourcing approaches in cancer research, and (3) published between January 2005 and June 2016. There were no limitations on the type of cancers included. Studies were excluded if they were grey literature such as dissertations or government reports, editorials, proceeding papers or other reviews.

The following data were extracted from each of the articles that met our criteria: (1) first author, (2) date of publication, (3) cancer type, (4) study objective, (5) size of the crowd, (6) length of time crowdsourcing was conducted, (7) recruitment platform, (8) incentives offered, (9) study outcome, and (10) potential limitations. The data extracted from these studies were explored and the findings were synthesized to identify common themes; however, due to the wide variation in the study objectives, designs, measures, and outcomes, the findings were not statistically combined into a meta‐analysis. Extracted data are displayed in Table 1.

**Table 1:** Extracted data from articles reviewed

Primary author, year, country	Cancer type	Study objective	Study outcome	Potential limitations	Dataset	Crowd size, length of time, recruitment platform, monetary incentive
Candido dos Reis (2015), UK, Brazil, The Netherlands, Spain Australia, Germany^13^	Breast	(1) To evaluate citizen scientists’ estrogen receptor (ER) classification and the association between ER status and prognosis by comparing their test performance against that of trained pathologists. (2) To enable the scoring of tumors labeled using immunohistochemistry by untrained members of the general public through an Internet‐based interface.	(1) Citizen scientists were able to classify ER expression in breast tumors with accuracy rate similar to that of trained pathologists (area under ROC curve for cancer cell identification: 0.95, 95% CI 0.94–0.96); area under ROC for ER status: 0.97, 95% CI 0.96–0.97). (2) Citizen scientists tended to overestimate the presence of cancer cells.	N/A	12,326 tissue microassays from samples from 6,378 patients in 10 studies	98,293 10/2012–6/2014 (20 months) Web‐based (Cell Slider) Media/news articles (Facebook, Reddit, UK television channel) None
Carter (2014), USA^20^	Ovarian	To examine public awareness and knowledge about ovarian cancer as compared with breast cancer by assessing a reasonable proxy of the US population through crowdsourcing using Amazon Mechanical Turk (AMT)	(1) Survey respondents consistently presented a lack of awareness of ovarian cancer impact or significance. (2) Survey respondents showed improved awareness among women (over men) and among those with increased knowledge of breast cancer and/or previous personal or family experience with cancer.	Survey respondents limited to US citizens with valid social security numbers, potentially falsely over‐representing Internet‐savvy and/or younger participants.	N/A	202 (of 232 eligible) 3/17/2013–3/25/2013 (8 days) Amazon Mechanical Turk $0.40 per completed survey
Eickhoff (2014), Switzerland^14^	Breast	(1) To explicitly compare the crowd‐powered expert to the individual performances of crowd or expert using a crowd of untrained workers to support medical experts. (2) To compare the effectiveness and efficiency of experts to that of crowd workers in detecting malignant breast cancer in medical images. Three separate research questions were evaluated.	The crowd was unable to outperform trained medical personnel in any of the investigated settings when used as a replacement for trained experts; however, untrained workers could support the work of experts, making it more efficient and less costly.	N/A	569 biopsy images	A total of 389 individual workers; each crowd varied in from *N* = 1 to *N* = 21 in multiple experiments designed to evaluate 3 separate research questions 1/2014–2/2014 Amazon Mechanical Turk and Crowd‐Flower $0.05/image
Ewing (2015), USA, Australia, Canada^23^	Unspecified	To identify the most accurate methods for calling somatic mutations in cancer genomes through evaluation of different approaches using crowdsourcing.	Teams routinely improved overall performance, especially in precision and especially with initial performance estimates, suggesting that studies may benefit from a multistep procedure.	Significant computational demands involved in aggregating multiple algorithms to enhance quality of mutation calls	248 analyses of 3 *in silico* tumors created with the tool for simulating cancer genomes	21 teams (unclear how many/team) 157 days Specifics of recruitment not provided in this article None
Good (2014), USA^18^	Breast	(1) To test the hypothesis that knowledge linking expression patterns of specific genes to breast cancer outcomes could be captured from players of an open, Web‐based game. (2) To translate the knowledge of the players, along with their ability to process textual information, into a ranked list of genes for use in the development of predictors for breast cancer prognosis.	Gene sets provided comparable performance to gene sets generated using other methods, including those used in commercial tests	Tasks presented in The Cure were knowledge intensive, requiring a significant level of preexisting expertise or substantial commitment to learning prior to playing the game.	25 different genes	1077 players 9/2012–9/2013 The Cure (web‐based game) None specified
King (2013), USA^15^	Skin	To explore the potential of crowdsourcing as a component of a more comprehensive skin cancer prevention effort, this study evaluates whether collective effort outperforms individual effort in the context of visual identification of atypical nevi.	Collective effort overcame the limitations of individual effort and exhibited superior sensitivity (.90)	N/A	40 nevi images	500 participants Not specified Recruited from shopping mall $15/participant
Leiter (2014), USA^22^	Prostate	To evaluate the feasibility and utility of using an Internet‐based crowdsourcing platform to inform the design of a clinical trial exploring the use of an antidiabetic drug, metformin, in prostate cancer.	Four major and five minor protocol modifications were made, including modifications to eligibility criteria and study procedures.	(1) Tech‐savvy crowd may not be representative. (2) Input occurred over 6 weeks, possibly delaying initiation of clinical trial unless initiated early in development process. (3) Concern about divulging elements of protocol (intellectual property management). (4) Unable to track whether protocol changes ultimately improve efficiency of resulting clinical trial.	N/A	60 physicians/researchers and 42 patients/advocates Six weeks Secure, web‐based platform (Transparency Life Sciences) enabling input (closed‐ and open‐ended responses) regarding important design elements of a planned clinical trial None mentioned
Margolin (2013), USA, UK, Norway^17^	Breast	(1) To assess whether a crowdsourced community challenge would generate models of breast cancer prognosis commensurate with or exceeding current best‐in‐class approaches. (2) To determine whether predefined performance criteria, real‐time feedback, transparent sharing of source code, and a blinded final validation dataset could promote robust assessment and improvement of breast cancer prognostic modeling approaches.	(1) Models submitted by challenge participants quickly exceeded the performance of a baseline model and steadily improved over time, though noted improvement was modest compared to baseline model. (2) Using the model, overall survival of breast cancer pts with aggressive tumors is harder to predict.	Intentional simplification of model and analysis strategies to focus only on concordance index would need refinement to yield more nuanced and meaningful data in future studies.	1981 breast cancer samples	354 registered participants from more than 35 countries July–October 2012: Three phases over 4 months (orientation, training, and validation) The Sage Bionetworks–DREAM Breast Cancer Prognosis Challenge (Actual recruitment strategies were not specified in this article) None mentioned
McKenna (2012), USA^21^	Colorectal	To investigate human performance in classifying polyp candidates under different presentation strategies	(1) Distributed human intelligence improved significantly with the additional information provided by the candidate polyp over a single image (2) Distributed human intelligence is a powerful tool that will aid in the development of computer‐aided detection for CT colonography	(1) Only one radiologist served as the expert (2) Knowledge workers were only presented with detection identified by computer‐aided development system (3) problem of residual stool not described to knowledge workers in effort to simplify training	600 polyp candidates from 50 patients	160 independent knowledge workers Time unspecified Amazon Mechanical Turk $0.01 per human intelligence task plus $5 bonus to the best knowledge workers
Parry (2015), USA^24^	Survivorship care planning	To increase the use of publicly available shared measures to enable comparability across studies and to facilitate identification of strategies for implementing care planning (or barriers to that planning) for cancer survivors	Provided a space to connect researchers and practitioners in ways usually not possible, but demonstrated that barriers to data harmonization cannot be overcome from a social media perspective.	Small crowd	7 domains comprising 51 constructs for which there were 124 measures	79 unique users February‐August 2012 (6 months) National Institute of Cancer (NCI)'s Grid‐Enabled Measures database None
Santiago‐Rivas (2015), USA^19^	Skin	(1) To determine whether people could be differentiated on the basis of their sun protection belief profiles and individual characteristics. (2) To explore the use of a crowdsourcing web service for the assessment of sun protection beliefs	(1) Identified three distinct clusters of sun protection barriers and three distinct clusters of sun protection facilitators. (2) Significant associations between gender, age, sun sensitivity, and cluster membership were identified	Potential bias in interpretation required for identifying subgroups (replication across two samples and use of variables not included in clustering process were used to limit bias).	40 sun protection belief questions	461 participants One day in July 2014 Amazon Mechanical Turk $0.40/survey
Wagholikar (2013), USA^16^	Cervical	(1) To report the methodology used to evaluate and improve the Clinical Decision Support System (CDSS) with participation of multiple users and experts before clinical deployment. (2) To ensure that the recommendations of the CDSS are of sufficient accuracy to be acceptable and useful to the providers	(1) Mismatch between provider and CDSS recommendations in 75/169 cases. (2) Provider recommendations were suboptimal in 56/169 cases (20.1% more than CDSS).	Expert review only for cases of mismatch; reviewers were not blinded to source of recommendations (provider vs. CDSS) though they were blind to identity of providers	175 test cases from patients at Mayo Clinic	25 potential users of CDSS 4/12/12–5/4/12 Web‐based application deployed on institution's internal network None

## Results

**Figure 1:**
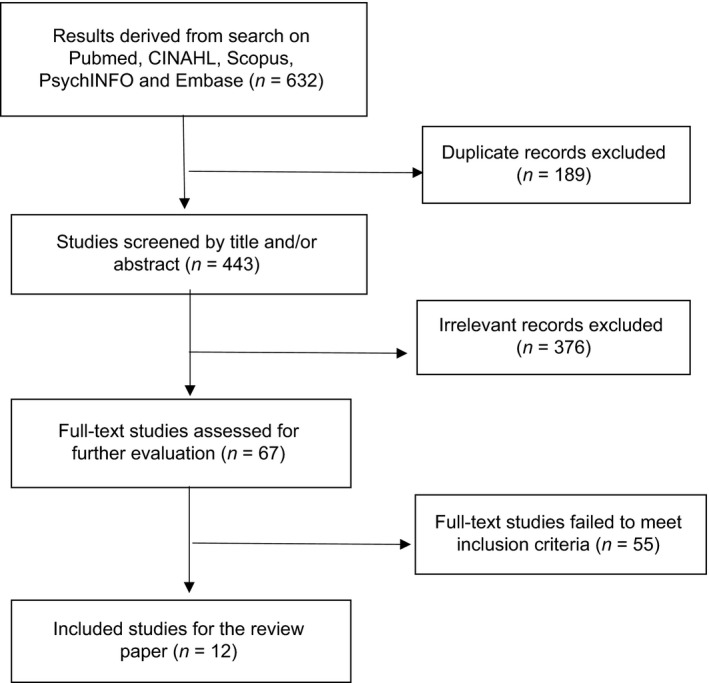
Flow diagram of the literature search.

Our literature searches using the search strings described above yielded 632 articles. Of these, 189 were duplicates, which left us with 443 articles, and 376 of these were eliminated based on the title and/or abstract of the article. The remaining 67 articles were subjected to a full‐text review by two raters (YL and JA), and 12 of these were found to meet the inclusion criteria and were retained for review. Please refer to the flowchart in Figure 1.

### General characteristics of the studies reviewed

All 12 articles retained for review describe findings from studies that featured various applications of crowdsourcing approaches to cancer research. Although our inclusion criteria could accommodate articles published between 2005 and June 2016, no studies published prior to 2012 were identified as meeting criteria. The primary authors for 10 of the studies were based in the United States; of the other two studies, one primary author was based in the United Kingdom 13, and the other in Switzerland 14. Because all of the studies except one 15 were conducted online, participants could be recruited worldwide; therefore, participants were not necessarily located in the same country as the researchers. Studies were conducted over time periods ranging from 1 day to 20 months, and crowd sizes (i.e., the number of participants) ranged from 25 participants 16 to 98,293 participants 13.

### Cancer types

The largest single type of cancer represented among these 12 studies was breast cancer, which was the focus of four (33%) of the studies reviewed 13, 14, 17, 18. Cancers of focus among six of the studies comprised skin 15, 19, ovarian 20, cervical 16, colorectal 21, and prostate 22. Of the remaining two studies, one used three simulated tumors with varying levels of cellular complexity and genetic mutation 23, while the other addressed the use of crowdsourcing for survivorship care planning following cancer 24.

### Study objectives

Due to its flexible nature, crowdsourcing lends itself to a wide range of applications, and this is reflected in the diversity of objectives represented among the studies that we reviewed, with two of the studies 18, 21 incorporating more than a single objective. With feedback/data coming from large numbers of people (i.e., the “wisdom of the crowd”) 9, crowdsourcing can be considered a strategy to reduce cost and increase efficiency without compromising the quality or accuracy of outcomes by replacing or augmenting the work of trained professionals, and six of the studies comprising this review 13, 14, 15, 18, 21, 23 investigated these potential uses of crowdsourcing. Moreover, crowdsourcing can be viewed as a way of harnessing input from a multitude of perspectives—both professional and nonprofessional—to accomplish goals such as developing clinical trials, assessment protocols, algorithms, and care plans and/or selecting candidate gene sequences for further investigation, and four articles in our review 16, 18, 22, 24 described studies of this nature. Three of these four studies 16, 22, 24 used targeted recruitment strategies aimed at stakeholders such as patients with the condition, advocates, treating clinicians, researchers, and/or potential end users of the modality in question, while the fourth 18 recruited from the general public but asked participants to identify their level of education and familiarity with biology and cancer; however, none of the studies specifically asked stakeholders to identify the nature of their investment in the problem, so it is possible that patients experiencing the condition being investigated might bring a different lens to the experience and the fact that this was not explicitly addressed may be regarded as a potential limitation of these studies. Finally, two studies that we reviewed 17, 18 addressed the use of crowdsourcing for prognosis or prognosis modeling, and two others 19, 20 involved crowdsourcing applications to assess the knowledge base and health‐related attitudes, beliefs, and behaviors of participants.

### Recruitment platform and incentives offered

Four studies that we reviewed 14, 19, 20, 21 utilized Amazon Mechanical Turk (MTurk) (https://www.mturk.com; Amazon Web Services, Amazon.com, Inc.) as a platform. Amazon MTurk is an Internet‐based crowdsourcing platform that allows users to distribute tasks to a large number of participants 21, 25.These studies offered small monetary incentives to participants ranging from $0.01/task (e.g., classifying potential colorectal polyps) 21 to $0.40 for completing a survey 19. Five studies that we reviewed 13, 17, 18, 22, 23 featured homegrown web‐based games or applications, and two studies were conducted using online platforms sponsored by either private industry 16 or government 24. Of these seven studies, none reported providing monetary incentives to participants. A single study 15 was conducted in a shopping mall and featured the face‐to‐face recruiting of 500 participants who each received a $15.00 incentive for completing a task involving the visual identification of atypical nevi (i.e., birthmarks).

### Study outcomes

#### Replacing or augmenting the work of trained professionals

Of the studies designed to evaluate the role of crowdsourcing to either replace or augment the work of experts 13, 14, 15, 18, 21, 23 (including comparing the effectiveness and/or accuracy of crowdsourced findings with those of trained experts), the findings were mixed. For example, Candido dos Reis et al. 13 demonstrated that crowdsourced participants, also known as citizen scientists, had accuracy rates similar to those of trained pathologists when identifying cancer cells and classifying estrogen receptor expression in breast tumors in 12,326 tissue microassays from 6,378 patients in 10 studies. In contrast, the two‐step human computation approach reported by Eickhoff 14 demonstrated that a crowd was unable to outperform trained medical professionals in identifying malignant breast cancer in 569 biopsy images. Nonetheless, Eickhoff 14 did show that trained experts completed biopsy evaluations faster and more reliably when images had been previously annotated by crowdsourced workers, suggesting that the crowd was effective in supporting the work of experts in a manner that could decrease cost while improving efficiency and enhancing accuracy.

Good et al.'s 18 game with a purpose (GWAP) asked crowdsourced participants to identify gene sets that could serve as prognostic predictors for breast cancer survival by reliably distinguishing between two groups of breast cancer patients: those who survived more than 10 years following diagnosis, and those whose survival was less than 10 years. Findings from this study demonstrated that players with previous knowledge and/or expertise in cancer biology were able to complete tasks more successfully than inexperienced players. Similarly, Ewing et al. 23 described their study of crowdsourced participants who responded to a challenge to detect and identify somatic gene mutations of varying levels of complexity. This study demonstrated ways in which crowdsourcing is useful in generating large datasets that can contribute to understanding error profiles in detecting somatic mutations, leading to refinements in the algorithms used. McKenna et al. 21 showed that the input of crowdsourced workers improved substantially with the addition of multiple images of colorectal polyps, demonstrating that crowdsourcing could play a valuable role in refining computer‐aided diagnostic systems to improve sensitivity and specificity in diagnosis of colorectal polyps by expert radiologists. The findings of the King et al. 15 skin self‐examination (SSE) study indicated that crowdsourced participants are better at detecting suspicious nevi than individuals conducting SSE. The study suggested that crowdsourcing approaches could be incorporated into larger multi‐component interventions to improve behavioral outcomes of SSE such as seeking skin cancer screening from a dermatologist.

#### Developing RCTs, protocols, applications, and/or care planning

The four studies 16, 21, 22, 24 examining the role of crowdsourcing for developing randomized controlled trials (RCTs), protocols, applications and/or care planning reflected the value of harnessing the power of large groups to develop or refine tools that can be used for clinical decision‐making or patient care while also noting some of the inherent limitations of these methods. Wagholikar et al. 16 described the use of crowdsourcing to enlist feedback from potential end users of a clinical decision support system (CDSS) to improve cervical cancer screening and surveillance recommendations by clinicians. The results contributed to refinements of the CDSS algorithm that in turn led to improved accuracy. McKenna et al. 21 showed that a decision support application used to identify suspicious colorectal polyps could be refined through input from crowdsourced workers. Leiter et al. 22 demonstrated the use of crowdsourcing to refine the protocol for a clinical trial by eliciting input from physicians, researchers, patients, and advocates recruited from a web‐based platform regarding the design of a clinical trial of metformin in prostate cancer. Using feedback from these potential stakeholders, modifications were made to the original protocol to address issues such as patient and physician awareness and acceptance of clinical trials. Parry et al. 24 report the findings of a technology‐mediated social participation trial in which the collective knowledge of clinicians, researchers, advocates, and policymakers involved in survivorship care planning was harnessed to develop an overarching framework to guide evidence‐based survivorship care planning and to identify and standardize process and outcome measures. This innovative approach provided a forum for connecting researchers and clinicians who would otherwise not have opportunities to collaborate, despite their shared goal of increasing consistency among the domains of survivorship care planning.

#### Developing cancer prognosis models

Two studies 17, 18 involved the use of crowdsourcing for prognosis modeling, and both supported the role of the crowd in achieving results equal or superior to the best currently available models. Margolin et al. 17 examined the use of a crowdsourced community to generate models to predict overall survival (OS) of patients with breast cancer. The study findings demonstrated that crowdsourced predictions of OS outperformed the best‐in‐class approaches available at the time of the study for all but the highest‐grade tumors with large numbers of positive lymph nodes. Good et al.'s GWAP 18 also used crowdsourcing for prognosis modeling. This study found that while participants with expert knowledge (i.e., PhD or MD) in biology and/or cancer produced gene sets with superior prognostic ability to that of nonexpert participants, none of the gene sets produced in the game was able to provide prognostic modeling for breast cancer survival that exceeded currently established sets derived through traditional means.

#### Assessing health‐related knowledge, beliefs, and behaviors

Two studies 19, 20 assessed the health‐related knowledge, beliefs, and behaviors of participants***.*** In the first of these studies, which assessed public awareness of and knowledge about ovarian cancer, including symptoms, risk factors, and prognosis/lethality, Carter et al. 20 demonstrated that a sample recruited with the MTurk platform could serve as a reasonable proxy for the general U.S. population. Participant responses reflected limited knowledge and awareness about ovarian cancer, though respondents with a personal or family history of breast and/or ovarian cancer had higher knowledge levels than those with no exposure to breast or ovarian cancer. In the second study, Santiago‐Rivas et al. 19 reported the findings of a study that also used MTurk to recruit participants who performed a task designed to assess beliefs and behaviors related to sun exposure protection, a powerful primary prevention strategy for avoiding skin cancer. These authors concluded that their crowdsourced participants were representative of a large portion of the general population—a portion that might be particularly difficult to target effectively when designing sun protection educational interventions.

## Discussion

The studies reviewed here reflect a growing interest in applying crowdsourcing approaches in cancer research. The purpose of this systematic review was to identify applications of crowdsourcing approaches in cancer research, to characterize the ways in which these approaches enhanced or hindered the research endeavor, and to explore potential future applications of crowdsourcing in cancer research. Our results suggest that crowdsourcing approaches are potentially applicable to cancer research across the continuum from prevention, diagnosis, prognosis, treatment, and survivorship.

We identified 12 studies that applied crowdsourcing approaches to cancer research to accomplish a range of goals that included (1) replacing or augmenting the work of trained professionals, (2) harnessing input from a multitude of perspectives to accomplish tasks such as developing clinical trials, assessment protocols, algorithms, and/or care plans, and selecting candidate gene sequences for further investigation, (3) developing cancer prognosis models, and (4) assessing the knowledge base and health‐related attitudes, beliefs, and behaviors of participants. We found that the applications of crowdsourcing represented in the studies reviewed here mirrored the traditional uses of crowdsourcing for distributing the burden of experts 13, 14, 16, solving complex problems 16, 20, 21, 22, 23, generating new knowledge 16, 18, 21, 22, and effectively sharing knowledge 19, 20, 24. Although none of these studies demonstrated a novel use of crowdsourcing as applied to cancer research, this is a typical finding in the early stages of adoption of a new technology or approach 26, 27.

One of the benefits of using crowdsourcing is its lower recruiting cost compared to traditional recruitment strategies, and that was borne out in this review. The costs of recruitment described in these studies ranged from $0.01/participant to $15.00/participant, which is substantially less expensive than the cost of recruiting and compensating participants in traditional research studies that did not use crowdsourcing. Only one study that we reviewed recruited a large group of participants (*n* = 500) in‐person from a shopping mall; the remainder of the studies recruited participants from the Internet. Although the shopping mall study 15 adopted a crowdsourcing approach (i.e., recruiting a large number of participants to leverage “the wisdom of the crowd”) 9, King et al. 15 demonstrated that in‐person recruiting of participants was more expensive than Internet recruiting. An additional benefit of using crowdsourcing in studies is the ability to recruit a large number of participants within a short amount of time, which also leads to cost savings. For example, Santiago‐Rivas et al. 19 recruited 461 participants in a single day, and Carter et al. 20 recruited 232 participants within a week. In fact, few of the studies that we reviewed were conducted over a long duration of time, the major exception being the Candido‐Reis et al. study 13, which gathered more than 98,000 responses over 20 months. Based on the findings of this review, we anticipate that low cost and ease of recruitment as well as the potentially accelerated timeframe of recruitment (and, consequently, data collection/study length) will be among the most attractive aspects of crowdsourcing in reducing burden to both cancer researchers and funding bodies.

One third of the studies (i.e., *n* = 4) 14, 19, 20, 21 that we reviewed adopted MTurk, while the others used homegrown platforms. Compared to homegrown platforms, the benefits of MTurk include ease of recruitment of participants from all over the world, inexpensive compensation (usually less than $1.00 for short tasks), supportive infrastructure, reliability, and subject prescreening functions. Although these authors 19, 20 have concluded that participants recruited through MTurk serve as a reasonable proxy for the population at large, at least one recent publication challenges this assumption 28, suggesting that researchers designing crowdsourcing protocols should thoughtfully consider the sociodemographic and political factors that may influence the suitability of MTurk respondents for their particular research question(s).

MTurk users generally are young and highly educated, which could lead to shortcomings of generalizability among cancer studies of underserved or aging populations. Therefore, cancer researchers should consider their target populations carefully when choosing a given crowdsourcing platform.

Although few of the studies demonstrated that collective knowledge from the general public could outperform experts, most studies showed that data generated by crowdsourcing was at least comparable to that generated by experts and could therefore be used to augment the work of experts—particularly when researchers have limited resources and/or the volume of data is high. For example, Eickhoff 14 revealed that the general population was unable to outperform experts in identifying malignant breast cancer from biopsy images. In contrast, Good et al. 18 demonstrated that their participants were able to identify genes implicated in breast cancer; however, the majority of these participants had some expertise in cancer biology. Additionally, although Ewing et al. 23 demonstrated similar success in gene identification among a sample of crowdsourced participants, cancer researchers—and researchers in general—should carefully consider the optimal applications of crowdsourcing to the specific needs of their research programs.

### Implications

The results of the studies included in this review provide a solid basis for recommending that cancer researchers consider ways to harness the power of these novel and innovative crowdsourcing approaches by incorporating them into future studies. Cancer researchers are often unaware of crowdsourcing approaches and the potential benefits they offer and therefore may not consider incorporating these methods when designing cancer research studies. Therefore, it is critical to increase the visibility and accessibility of crowdsourcing methods, perhaps by providing online educational offerings to cancer researchers. Additionally, the findings of our review suggest the importance of helping researchers understand the ways in which crowdsourcing might provide them with access to participants whose education and/or expertise could substantially reduce or augment the work of costly trained experts. Access to such populations of participants could be accomplished through targeted recruitment strategies informed by crowdsourcing principles. Finally, as routine access to the Internet continues to diffuse across socioeconomic and cultural barriers, the degree to which crowdsourced participant populations will mirror populations of interest is likely to increase, making crowdsourcing an even more feasible, practical, affordable, and relevant addition when designing cancer research. This innovative approach provided a forum for connecting researchers and clinicians who would otherwise not have opportunities to collaborate, despite their shared goal of increasing consistency among the domains of survivorship care planning.

### Limitations

This systematic review was a qualitative systematic review—not a meta‐analysis. As such, our review of the literature was designed to generate neither effect sizes nor other aggregate metrics. Nevertheless, given our interest in identifying and exploring the types and breadth of current crowdsourcing applications in cancer research, the qualitative systematic review methodology that we followed offered the most appropriate lens. Additionally, because the literature reviewed considered studies published (1) in English only, (2) during only the years 2006–2016, and (3) in only a limited group of databases (*n* = 5), our results may reflect potential selection bias, which must be taken into consideration.

## Conclusion

In this systematic review, we analyzed and summarized studies to identify the current range of applications of crowdsourcing approaches in cancer research. Despite its limitations, crowdsourcing possesses tremendous potential to improve the quality and speed of certain types of cancer research while reducing costs. Findings in the studies included in this review could be applied to the cancer research in various ways, providing researchers access to experts to aid in study design or protocol development, crowds to augment the work of trained experts, and/or actual participants in randomized control trials. Due to the rapidly changing nature of the Internet, longitudinal studies tracing trends in the optimal uses of crowdsourcing in cancer research over time would be fruitful additions to the continued efforts to refine the applications of crowdsourcing approaches in academic scholarship. Additionally, widespread efforts to disseminate knowledge about crowdsourcing as a modality and to connect researchers from the various disciplines within health care with those from information technology and computer science are likely to yield increasingly novel approaches to persistent challenges through cross‐pollination between research areas that have traditionally been siloed and therefore distinct.

## Conflict of Interest

The authors report no conflicts of interest.
